# Wing sexual dimorphism of pathogen-vector culicids

**DOI:** 10.1186/s13071-015-0769-6

**Published:** 2015-03-14

**Authors:** Flávia Virginio, Paloma Oliveira Vidal, Lincoln Suesdek

**Affiliations:** Laboratório de Parasitologia, Instituto Butantan, São Paulo, SP Brasil; Programa de Pós-Graduação em Biologia da Relação Patógeno-Hospedeiro, Instituto de Ciências Biomédicas, Universidade de São Paulo, São Paulo, SP Brasil; Programa de Pós-Graduação em Medicina Tropical, Instituto de Medicina Tropical, Universidade de São Paulo, São Paulo, SP Brasil

**Keywords:** *Culex*, *Aedes*, *Anopheles*, *Ochlerotatus*, Culicidae, Mosquitoes, Geometric morphometrics

## Abstract

**Background:**

Sexual dimorphism in animals has been studied from different perspectives for decades. In 1874 Darwin hypothesized that it was related to sexual selection, and even after nearly 140 years, when additional empirical data has become available and the subject has been investigated from a contemporary viewpoint, this idea is still supported. Although mosquito (Culicidae) wings are of great importance as they play a sex-specific role, little is known about wing sexual dimorphism in these pathogen-vector insects. Detection and characterization of wing sexual dimorphism in culicids may indirectly enhance our knowledge of their epidemiology or reveal sex-linked genes, aspects that have been discussed by vector control initiatives and developers of genetically modified mosquitoes.

**Methods:**

Using geometric morphometrics, we carried out a comparative assessment of wing sexual dimorphism in ten culicid species of medical/veterinary importance from genera *Culex, Aedes, Anopheles* and *Ochlerotatus* collected in Brazil.

**Results:**

Discriminant analysis revealed significant sexual dimorphism in all the species studied, indicating that phenotypic expression of wing shape in mosquitoes is indeed sex-specific. A cross-validated test performed to reclassify the sexes with and without allometry yielded very similar results. Mahalanobis distances among the ten species showed that the species had different patterns of shape sexual dimorphism and that females are larger than males in some species.

**Conclusion:**

Wing morphology differed significantly between species. The finding of sexual dimorphism in all the species would suggest that the wing geometry of Culicidae is canalized. Although sexual dimorphism is prevalent, species-specific patterns occur. Allometry was not the main determinant of sexual dimorphism, which suggests that sexual selection or other evolutionary mechanisms underlie wing sexual dimorphism in these insects.

## Background

Typically, morphological sexual dimorphism (SD) is dichotomically classified into size and shape dimorphism; however, there is also known to be interaction between body size and shape (allometric SD). Although in some cases SD appears to be the result of allometry [1], examples of SD that can be attributed purely to size [2] or shape [3] have also been reported. For decades, SD in animals has been investigated from either an evolutionary or ecological point of view. About 140 years ago, Darwin [4] hypothesized that SD might be related to sexual selection, i.e., the process whereby the maintenance of sex-specific traits is driven by the preference of the opposite sex. Contemporary views of SD take into account empirical data and new constraints and concepts but preserve the underlying idea behind Darwin’s hypothesis [5].

Insects have been used as models for investigating SD as sex-related differences can occur in various organs, such as the eyes [6], legs [7,8] and head capsule [9]. Other structures and features, such as hairs on the antennae, body size, mouthparts, genitalia [10] and wings [11,12] can also be observed and help with sexing. Insect wings may have evolved through selective action associated with mating behavior [13,14]. Sexual behavior, such as courtship songs, can depend on sex-specific wing geometry [15].

Few studies have been published to date on wing SD in Culicidae, although this family includes insects which are vectors of etiologic agents of serious human diseases. Wing SD in this family has been described occasionally in taxonomic keys, but its specific patterns and variability are not well known. Wing sexual size dimorphism (SSD) and sexual shape dimorphism (SShD) have already been reported in *Oc. scapularis* [11], but a comparative perspective has yet to be explored. Investigation of wing SD in culicids may indirectly enrich our knowledge of their epidemiology given that both the epidemiological relevance of and role played by wings are particular to each sex. Only females are hematophagous and pathogen-competent and use their wings to ensure an accurate approach to other animals and suck their blood, while males can copulate with several mates and use wing beats to attract the opposite sex during courtship. Wing SD merits study because it may underlie sex-linked genetic markers, which are especially useful for the development of genetically-modified mosquitoes [16,17].

An initial approach to the study of wing SD in Culicidae would be to detect and quantify wing SD in species that are representative of the main taxonomic subgroups. This would yield information about the frequency of SD and subsequently provide insights into the role of phylogenetic constraints and/or species-specific adaptation in the evolution of SD. Nowadays, investigation of wing SD in many samples is more feasible than in the past because of the availability of geometric morphometrics, a cheap and highly accurate technique that has become increasingly popular [11,18-33].

We decided to investigate whether male and female culicids have different wing characteristics, a hypothesis that was proposed following a case study of *Oc. scapularis* [11]. We also hypothesized that different species of Culicidae have different degrees of SD, as has been reported for Drosophilidae [1]. To this end we investigated ten Culicidae species of medical/veterinary importance from the two main subfamilies of Culicidae using geometric morphometric analysis of wing shape and wing size separately.

## Methods

### Collection of the biological samples

Mosquitoes of the genus *Aedes (Aedes aegypti, Aedes albopictus), Anopheles (Anopheles albitarsis l.s., Anopheles cruzii, Anopheles homunculus, Anopheles strodei l.s., Anopheles triannulatus l.s.)*, *Culex (Culex quinquefasciatus, Culex nigripalpus)* and *Ochlerotatus (Ochlerotatus scapularis)* were collected between 2007 and 2013 in the States of São Paulo and Minas Gerais (Table 1). The immature stages (eggs, larvae and pupae) were collected from artificial and natural breeding sites and maintained in the laboratory under standard temperature and humidity conditions (25 ± 1°C; 80 ± 10%) with a photoperiod of 12:12 (light:dark) until the emergence of adult mosquitoes. Adult mosquitoes were collected with entomological aspirators and white Shannon-type traps. All individuals were identified at the species level [10,34-37] and stored in 70% ethanol.

**Table 1:** **Data for Culicidae species collected**

**Species**	**Geographic location**	**Geographic coordinate**	**Year**	**No. of specimens**	**Stage**	**Type of breeding**
*Cx. quinquefasciatus*	Rio Pinheiros, SP	-23.595138 S; -46.694258 W	2007	17 F / 17 M	Adult	Natural
*Cx. nigripalpus*	Parque Ecológico do Tietê, SP	-23.480094 S; -46.509274 W	2007	16 F / 20 M	Adult	Natural
*Oc. scapularis*	Tremembé, SP	-22.954916 S; -45.543534 W	2010	24 F / 25 M	Adult	Natural
*Ae. aegypti*	São José do Rio Preto, SP	-20.810039 S; -49.368546 W	2011	25 F / 25 M	Immature	Artificial
*Ae. albopictus*	Campinas, SP	-22.905906 S; -47.069657 W	2011	25 F / 25 M	Immature	Artificial
*An. albitarsis l.s.*	Frutal, MG	-20.030129 S; -49.021425 W	2013	15 F / 09 M	Immature	Natural
*An. homunculus*	Cananéia, SP	-24.695063 S; -47.870972 W	2012	32 F / 24 M	Immature	Natural
*An. triannulatus l.s.*	Frutal, MG	-20.030129 S; -49.021425 W	2013	22 F / 17 M	Adult and Immature	Natural
*An. strodei l.s.*	Frutal, MG	-20.030129 S; -49.021425 W	2013	22 F / 16 M	Immature	Natural
*An. cruzii*	Cananéia, SP	-25.012376 S, -47.935381 W	2012	40 F / 33 M	Immature	Natural

S: South, W: West, F: Female, M: Male.

### Sample preparation

Wings were detached from the thorax of each individual (both males and females) and mounted between a slide and coverslip as described by Lorenz et al. [23]. Images of the wings were captured by a Leica DFC320 digital camera coupled to a Leica S6 stereoscope at 40X magnification.

**Figure 1:**
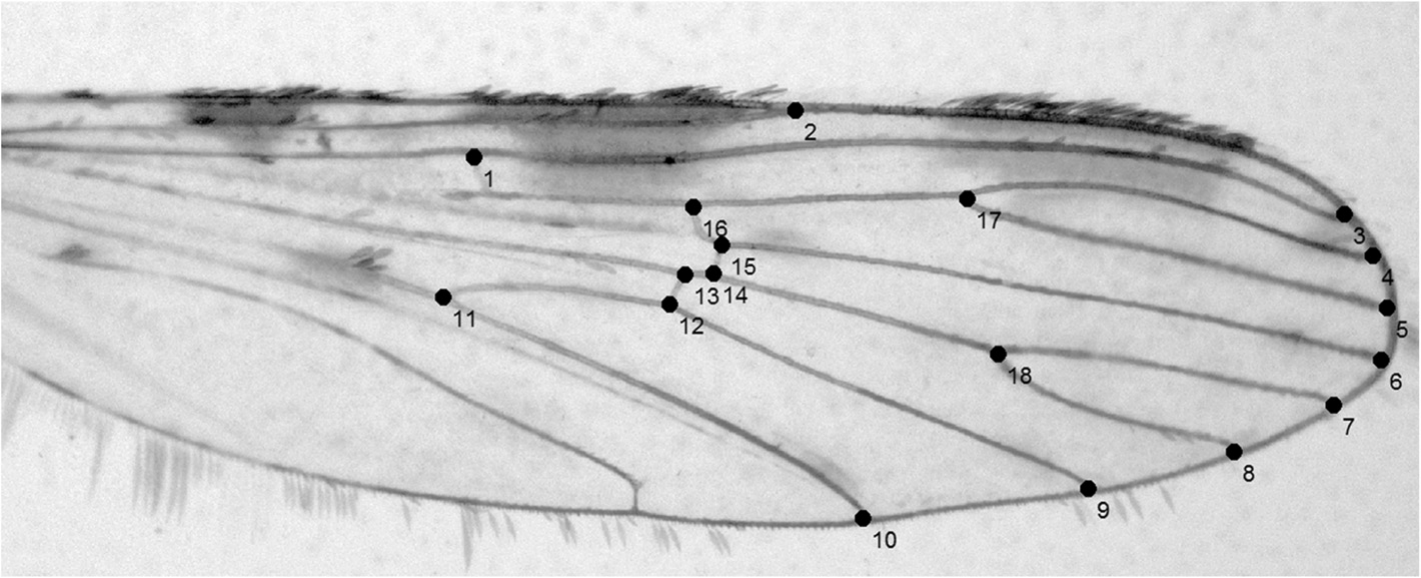
**Right wing of** ***Anopheles strodei l.s.*** **with the 18 landmarks used in this study.**

The coordinates of eighteen landmarks at the vein intersections on these images were digitized using TpsDig v. 1.4 software [38]. This set of landmarks has proven to be sufficiently sensitive to describe SD [11] (Figure 1).

Most of the analyses were performed with right wings; left wings were only used when the corresponding right wing was damaged.

### Morphometric analyses

To analyze the contribution of size and shape to SD separately, we removed the allometric effect in all the analyses. Wing shape and size SD was determined in the different species. Morphological similarity among the ten species sampled was also examined. This was done separately for males and females.

Variations in wing shape (partial warps) were determined by Procrustes superimpositions through generalized least squares, eliminating the differences in orientation, position and isometric size [38]. Multivariate regression of Procrustes coordinates versus centroid size (CS) was used to remove allometric effects, and a permutation test with 10,000 randomizations was applied using MorphoJ software [39] to test the significance of the allometry. Differences in wing shape SD were determined by canonical variate analysis using MorphoJ software. Wing shape dissimilarity between females and males of each species was estimated by Mahalanobis distances (MD) and compared in a permutation test with 10,000 randomizations using MorphoJ software.

Morphological divergence among the samples was illustrated by UPGMA phenograms constructed using PHYLIP (Phylogeny Inference Package) v.3.6 distributed by J. Felsenstein [40], Department of Genome Sciences - University of Washington, Seattle/WA). To test for dissimilarity between males and females, individuals were reclassified according to their similarity to each group using the MD.

The global size of the wing was estimated using CS, the square root of the sum of the squared distances of all landmarks from the centroid. GraphPad InStat v.3.01 (San Diego, CA) was used to compare CS scores between samples. The unpaired T-test was used in populations that had a Gaussian distribution and the Mann-Whitney test in populations that did not. The CS of all the species were represented graphically with the MOG v.82 program [41].

## Results

**Figure 2:**
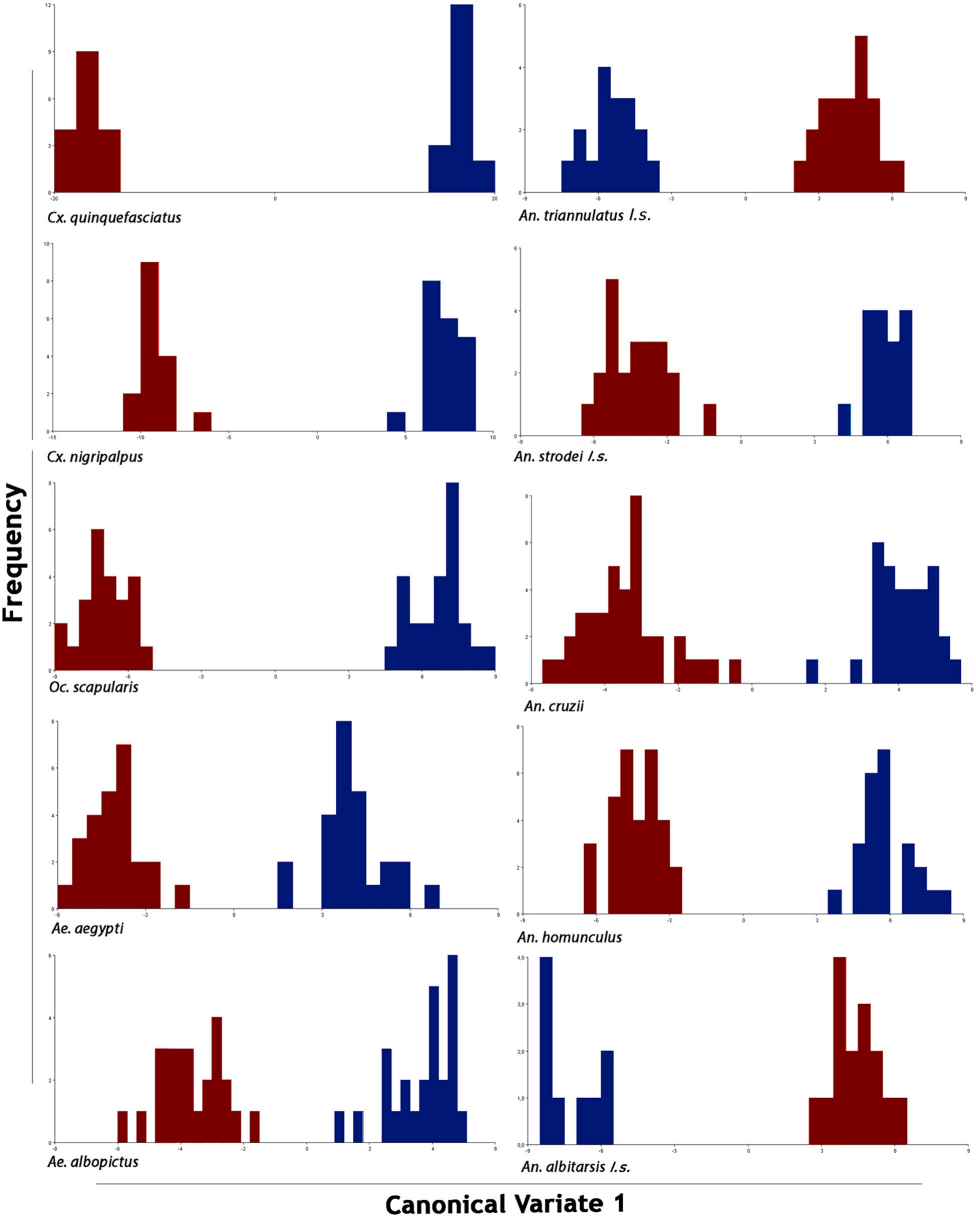
**Wing shape diagrams of first canonical variable from the comparison of males (blue) and females (red).**

We first removed the allometric effect from all the analyses as, although the study was a macroevolutionary one, we wanted to analyze shape and size separately. Canonical variate analysis revealed differences in wing shape between all the species analyzed for both males and females, thus we could see that all species have clearly sexually dimorphic wings (Figure 2). MD (without allometry) ranged from 7.34 to 34.06 (p < 0.0001), indicating a great variability in SD between species. *Ae. albopictus* and *Cx. quinquefasciatus* were the least and most sexually dimorphic species, respectively.

Table 2 shows the phenetic differentiation between sexes in each species, indicates that allometry was low (<10%) or not significant and did not contribute significantly to SShD (see the MD columns).

**Table 2:** **Phenetic differentiation between sexes in each of the species**

	**Number of individuals**			**Cross validation score**					**Mahalanobis Distance**		
**Species**			**Without allometry (%)**		**With allometry (%)**						
	**Females**	**Males**	**Females**	**Males**	**Females**	**Males**	**Allometry (%)**	***P***	**With allometry**	**Without allometry**	***p*** ^***†***^
*Cx. quinquefasciatus*	17	17	100	100	71	71	4.57	0.1454	34.10	34.06	**<.0001**
*Cx. nigripalpus*	16	20	62.5	75	75	65	8.51	**0.0025**	16.70	16.62	**<.0001**
*Oc. scapularis*	24	25	100	100	100	96	5.64	**0.0147**	13.71	13.71	**<.0001**
*An. albitarsis l.s.*	15	9	87	89	53.3	67	9.50	**0.0144**	8.70	11.74	**<.0001**
*An. homunculus*	32	24	97	96	100	96	7.08	**<.0001**	9.94	10.23	**<.0001**
*An. strodei l.s.*	22	16	59.1	81.3	59.1	81.3	5.91	**0.0219**	9.19	10.14	**<.0001**
*An. triannulatus l.s.*	22	17	68.2	58.8	68.2	70.6	5.37	**0.0379**	9.00	9.68	**<.0001**
*Ae. aegypti*	25	25	96	92	92	84	6.55	**0.0003**	8.68	8.16	**<.0001**
*An. cruzii*	40	33	87.5	94	92.5	100	5.20	**0.0001**	7.12	7.57	**<.0001**
*Ae. albopictus*	25	25	88	88	88	88	3.70	0.0689	6.78	7.34	**<.0001**

Signifiacant p-values are in bold. †: The p-values for the MD were equivalent for both cases (with/without allometry).

Scores for the reclassification test after validation; allometric residues; and Mahalanobis distances and their respective statistical significances.

**Figure 3:**
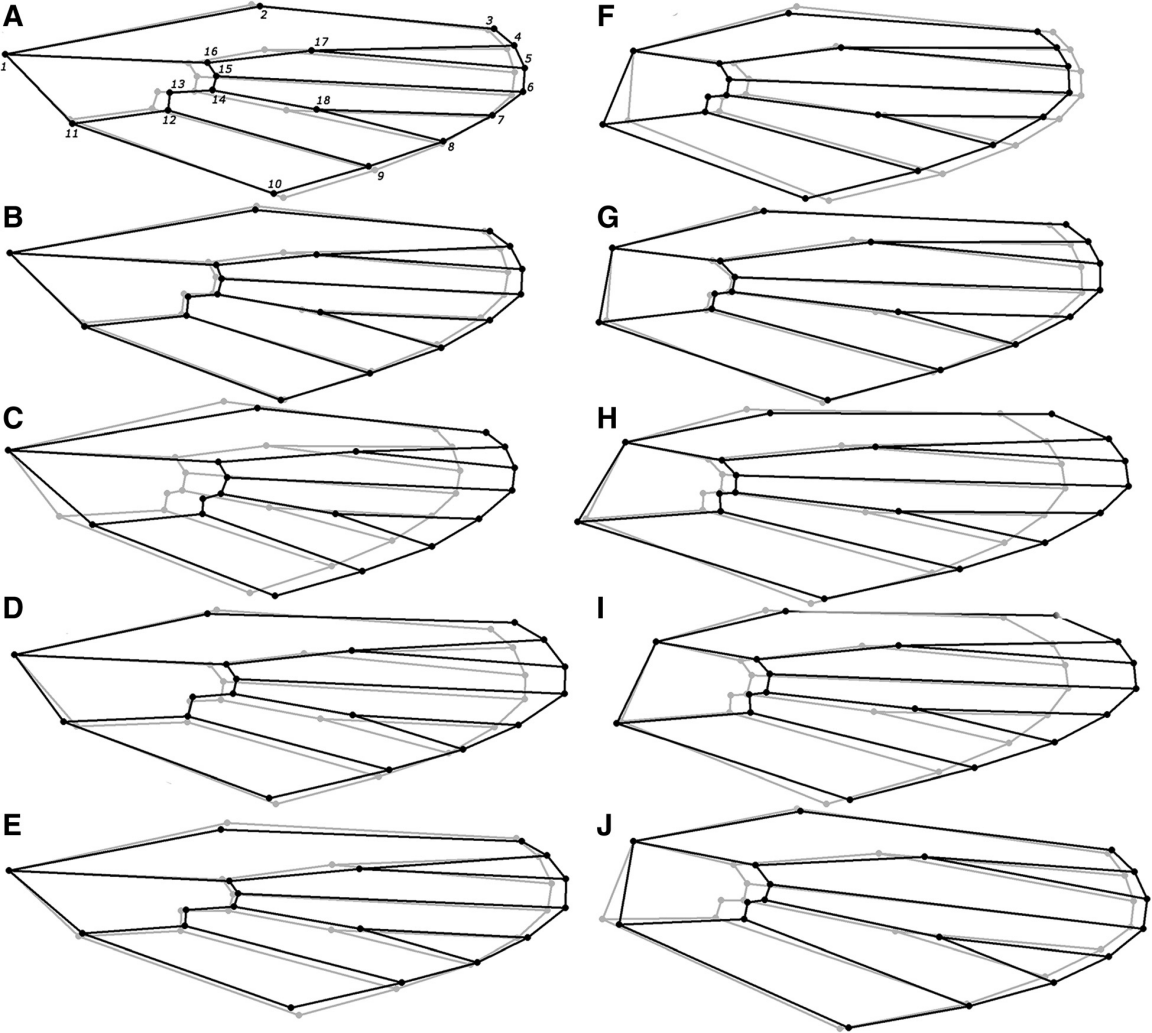
**Intraspecific extremes of differentiation.** Gray line: female, black line: male. In general the wings of females were wider and shorter than those of males. The most variable landmarks are in the proximal and distal regions of the radial and median veins. **A**: *Cx quinquefasciatus*; **B**: *Cx nigripalpus*; **C**: *Oc. scapularis*; **D**: *Ae. aegypti*; **E**: *Ae. albopictus*; **F**: *An. triannulatus l.s*.; **G**: *An. strodei l.s.*; **H**: *An. homunculus*; **I**: *An. cruzii*; **J**: *An. albitarsis l.s*. Shape variation: 1X. Diagrams were superimposed on landmark 1.

Comparison of the extremes of differentiation for males and females revealed major displacement of the landmarks between the sexes (Figure 3). As in *Drosophila* [1,42-44], the most variable landmarks were in the proximal and distal regions of the radial and medium veins. Females were slightly wider and significantly shorter than males.

**Figure 4:**
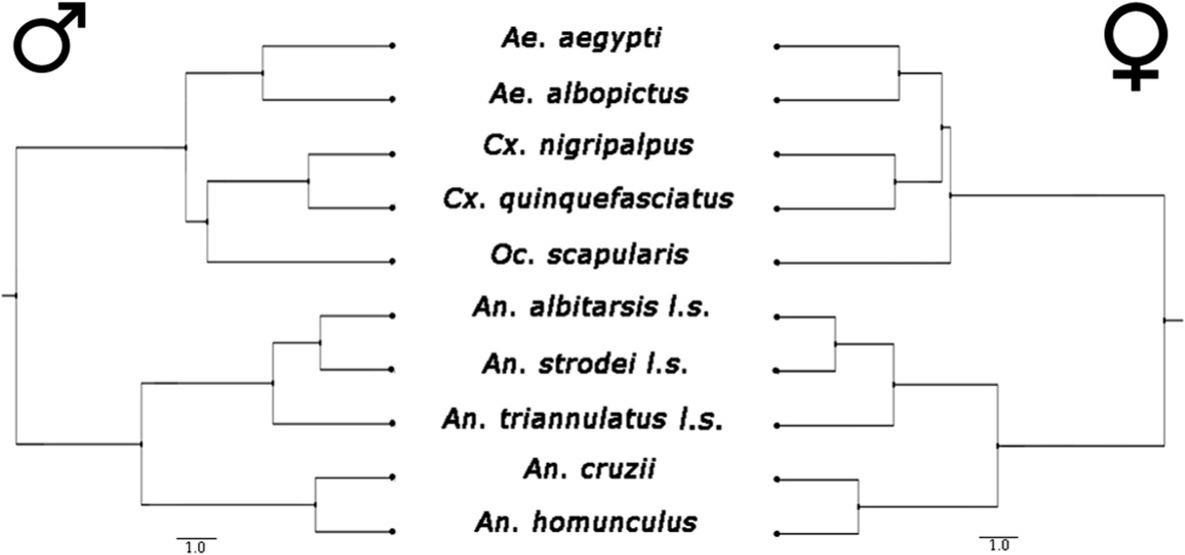
**UPGMA phenograms of MD for males and females separately.** The upper cluster corresponds to the Culicinae subfamily, and the lower cluster to the Anophelinae subfamily.

The phenograms of the pairwise distances for the ten species (sexes separated) corroborated the SD found in the other analyses. The “Anophelinae - males” cluster was symmetrical to the “Anophelinae - females” cluster, but clusters comprising Culicinae representatives were not congruent between the sexes (Figure 4).

**Figure 5:**
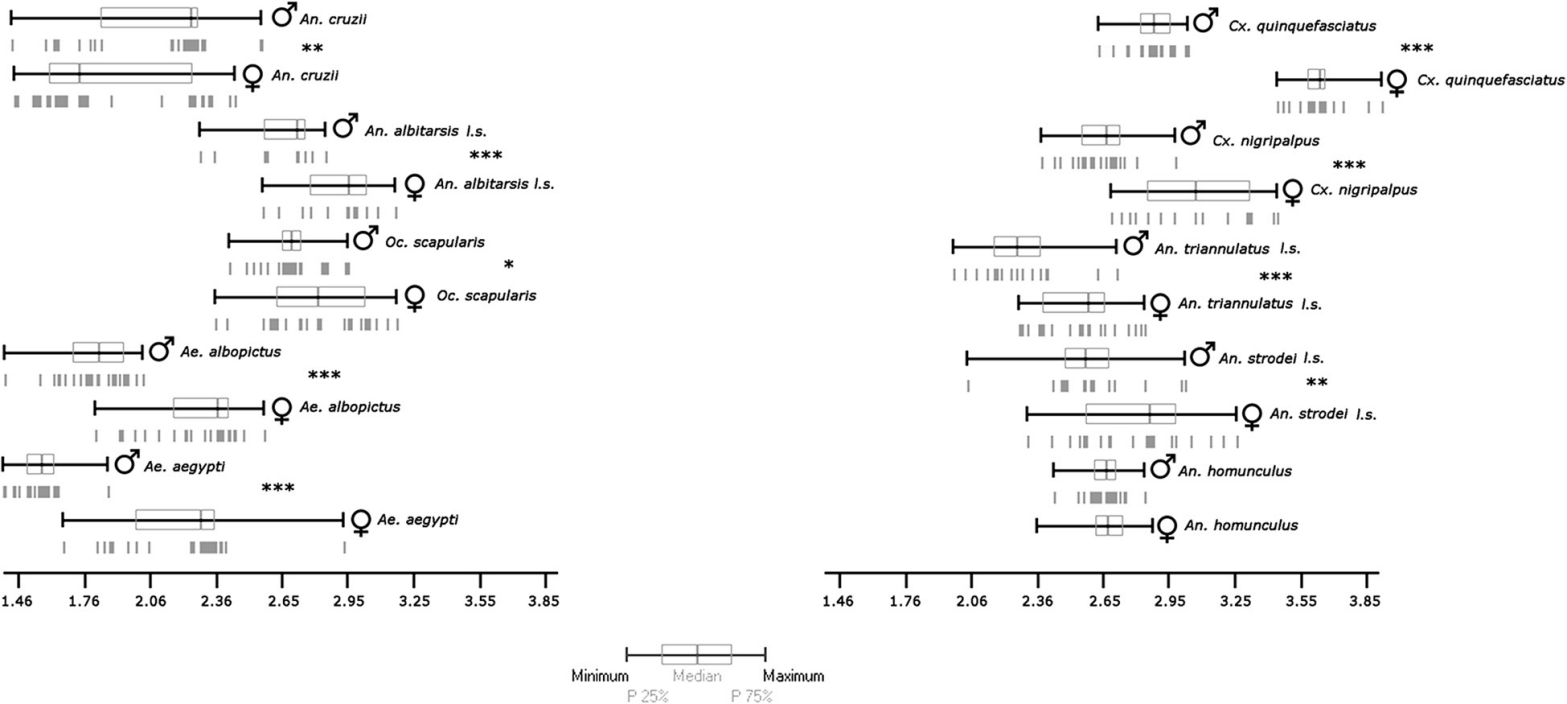
**Descriptive statistics of wing CS (in mm) of males and females from different species.** Vertical lines: individuals; *: significant; **: very significant; ***: extremely significant.

A cross-validated reclassification test based on the MD was performed with an accuracy of 53.3 to 100%. The same analysis was carried out taking into account the allometric effect to identify whether allometry contributed significantly to SD in the species studied. The score in this analysis was 58.8 to 100%. Both analyses (with and without the effects of allometry) yielded very similar results except for the species *Cx. quinquefasciatus, An. albitarsis l.s.* and *Ae aegypti*, which had high scores when allometric effects were included. Analysis of CS indicated SSD in all species except *An. homunculus* (Figure 5).

Wing CS for each species lay in the following ranges (in mm): *Ae. albopictus* (F: 1.85- 2.58, M: 1.46-1.91), *Cx. quinquefasciatus* (F: 3.40-3.85, M: 2.64-3.02), *Ae. aegypti* (F: 1.72-2.92, M: 1.46-1.91), *Cx. nigripalpus* (F: 2.69-3.40, M: 2.39-2.97), *An. triannulatus l.s.* (F: 2.30-2.83, M: 2.01-2.71), *An. strodei l.s.* (F: 2.33-3.23, M: 2.07-3.01), *An. albitarsis l.s.* (F:2.57-3.14, M:2.30-3.14), *Oc. scapularis* (F: 2.37-3.15, M: 2.43-2.94), *An. homunculus* (F: 2.37-2.87, M: 2.45-2.84), *An. cruzii* (F: 1.51-2.45, M: 1.49-2.57). The species with the lowest wing CS in males were *Ae. albopictus* and *Ae. aegypti,* while the corresponding species for females was *An. cruzii*. The species with the largest wing CS in males and females were *An. albitarsis l.s.* and *Cx. quinquefasciatus*, respectively.

**Figure 6:**
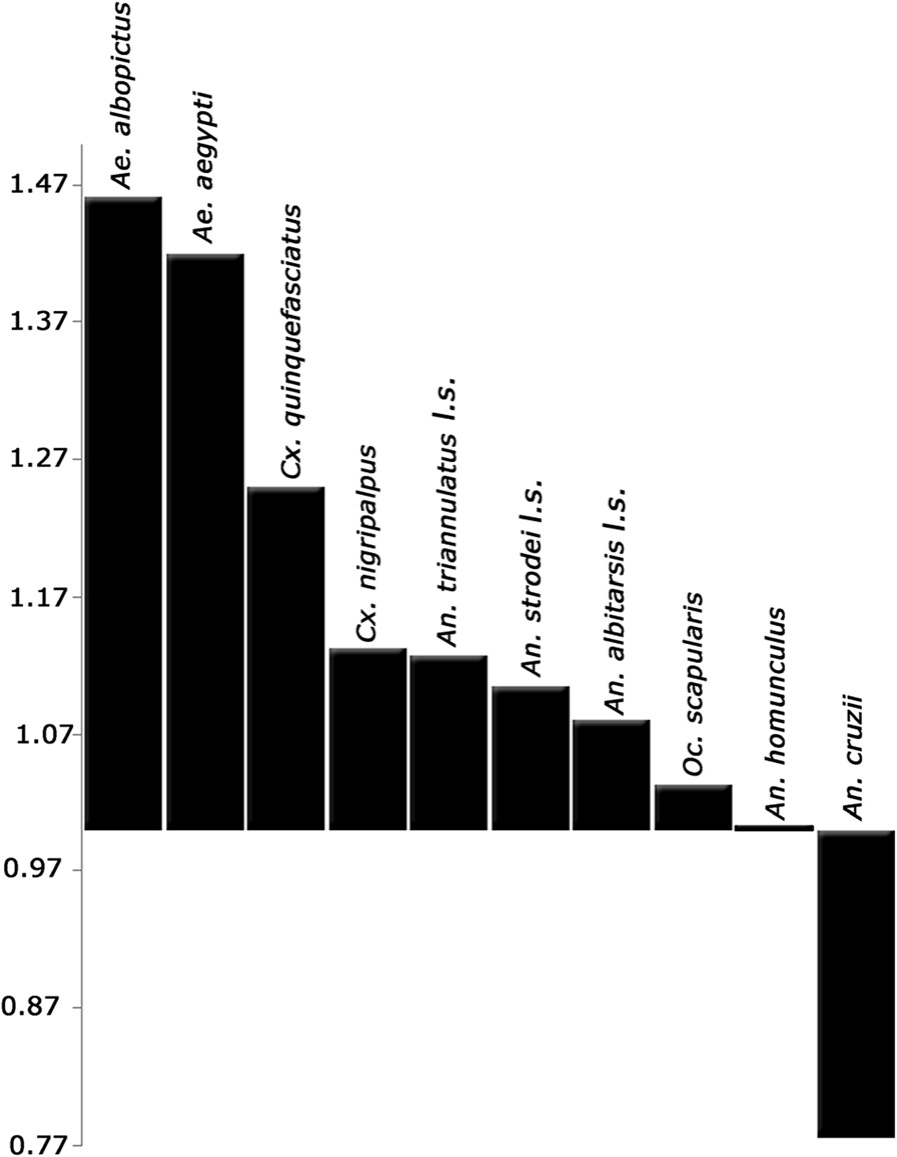
**Ratio of mean female CS to mean male CS for each species.**

The graphical representation of the ratio of mean female CS to mean male CS (Figure 6) shows that females were larger than males in most species. The exceptions were *An. homunculus* (ratio = 1), for which the analysis failed to reveal SSD, and *An. cruzii* (ratio = 0.78), for which females were smaller than males.

## Discussion

Our results indicated wing SD in the ten mosquito species analyzed, suggesting that the phenotypic expression of wing shape is sex-specific. This is in accordance with the findings reported by Devicari et al. [11] in *Oc. scapularis*.

There was significant SShD in all the taxonomic groups analyzed, showing a very marked pattern of SD in the family Culicidae. There may be some evolutionary canalization of wing shape that keeps wings sexually dimorphic in so many species, including even those that are phylogenetically more distant in the family. Such canalization does not appear to be driven by allometry because the allometric effects in all the species were low (and sometimes not significant) and only exerted a marginal influence on SShD. Because of the low allometric values observed (as previously detected in *An. cruzii* by Lorenz et al. [32]), we cannot assert that SD in culicids is only an allometric effect, as some authors have conjectured [22]. It is possible that the ubiquity of SShD is maintained by the different ways in which wings are used by each sex, i.e., the overall wing shape may be adapted to each sex-specific function.

Although, unlike for crickets [15], we do not know how wing shape determines wing beat dynamics in culicids, this interpretation has a parallel in the evidence of sex-specific selection of wing beats in some *Anopheles* species [45,46]. We cannot discard the possible existence of some sex-specific non-adaptive developmental constraint behind the supposed canalization.

The incongruence between Culicinae clusters in the phenograms suggests that species of this subfamily have different patterns of sex dimorphism. Accordingly, Culicinae is the most heterogeneous cluster in terms of the degree of SShD and SSD. As Culicinae is also the most macroevolutionarily derived taxon, the phylogeny of SD merits further investigation.

In some species of insects, males can be larger than females but have less body mass [47]. In our study, SSD was common to most species, although it manifested differently. In most of the species, the females were larger, as reported for other groups of insects such as *Ophion intricatus* [3], *Drosophila melanogaster* [48,49], *Cx. quinquefasciatus* [30], *Ae. albopictus* [28], *Stenurella melanura* [50], *Scapteriscus acletus* and *Scapteriscus vicinus* [51]. The fact that SSD did not follow the female > male pattern in *An. cruzii* and was not present in *An. homunculus* may be the result of evolutionary and environmental factors particular to these species, which are evolutionarily and ecologically closely related [23]. SSD is probably more variable and less canalized than SShD, i.e., size is evolutionarily less stable than shape. This corroborates the findings of Devicari et al. [11] and confirms the theory put forward by Dujardin [18] according to which wing size in insects may be plastic and more influenced by non-genetic factors. Curiously*, An. cruzii* also had low SShD (as well as “inverted” SSD), making it a particularly unusual species compared with the others. We do not yet know the causes of this variability. Although some studies suggest that SSD and SShD can be adaptive [1-3,39], a clear explanation for these types of SD has yet to be proposed.

Although we detected several features of wing SD in Culicidae species here, an explanation for the observed patterns has yet to be formulated. Considering that SD is prevalent and not primarily due to allometry, we believe that complex evolutionary mechanisms are responsible for the maintenance of SD in mosquitoes. The interspecific divergence of SD has also yet to be explained. It has been suggested that sexual selection and mating system are primary forces that direct the evolution of SD in insects [52], as occurs in Drosophilidae, the best known sister group of Culicidae. Although it has been suggested that ecological SD occurs, it does not appear to be the most important form of SD [13]. However, although many researchers support the idea that the evolution of SD is directed by sexual selection [9,53,54], others believe it may be an effect of natural selection [49]. Chenoweth et al. [55], in experimental studies of *Drosophila serrata*, found that SD tends to increase under the pressure of sexual selection and decrease under the pressure of natural selection. Nevertheless, as we have described here many peculiarities of wing SD in culicids, we do not know to what extent the considerations regarding other insects are generalizable to Culicidae.

Establishing answers to the following questions could help clarify whether SShD is driven by sexual selection: Does wing geometry influence wing beat and other sexually selectable wing traits? Which genes are involved in determining wing shape in culicids?

What is the inheritance and expressivity of these genes? Until recently, little was known about wing SD in Culicidae. It is now clear that this subject is an open field for further research that will eventually enrich our knowledge of the biology, evolution and epidemiology of these mosquitoes.

## Conclusion

We believe that wing characters provide useful information for the study of SD and are a practical tool for identifying the sex of culicids. Our findings of apparent canalization of SShD in all the species studied and high variability of SSD appear to reflect complex underlying evolutionary factors. Further, more detailed studies of the determinants of SD may provide essential information for an understanding of the biology of mosquitoes and subsequently help improve vector control methods.

## References

[1] Gidaszewski NA, Baylac M, Klingenberg CP. Evolution of sexual dimorphism of wing shape in the *Drosophila melanogaster* subgroup. BMC Evol Biol. 2009;9: doi:10.1186/1471-2148-9-110 10.1186/1471-2148-9-110 PMC2691407 19457235

[2] Fairbairn DJ, Blanckenhorn WU, Székely T. Sex, size and gender roles: evolutionary studies of sexual size dimorphism. Oxford Scholarship Online. 2007;doi:10.1093/acprof:oso/9780199208784.001.0001

[3] Benítez HA, Bravi R, Parra LE, Sanzana MJ, Sepulveda-Zuniga E (2013). Allometric and non-allometric patterns in sexual dimorphism discrimination of wing shape in *Ophion intricatus*: might two male morphotypes coexist. J Insect Sci.

[4] Darwin CR (1874). The descent of man, and selection in relation to sex.

[5] Fairbairn DJ (1997). Allometry for sexual size dimorphism: pattern and process in the coevolution of body size in males and females. Annu Rev Ecol Syst.

[6] Meyer-Rochow VB, Reid WA (1994). Male and female eyes of the Antarctic midge *Belgica antarctica* (Diptera, Hironomidae) - a scanning electron microscope study. Appl Entomol Zool.

[7] Adler PH, Adler CRL (1991). Mating behavior and the evolutionary significance of mate guarding in 3 species of crane flies (Diptera: Tipulidae). J Insect Behav.

[8] Eberhard WG (2002). Physical restraint or stimulation? The function(s) of the modified front legs of male *Archisepsis diversiformis (Diptera, Sepsidae)*. J Insect Behav.

[9] Wilkinson GS, Dodson GN (1997). Function and evolution of antlers and eye stalks in flies.

[10] Forattini OP. Culicidologia médica, vol. 2. 2002

[11] Devicari M, Lopes AR, Suesdek L (2011). Wing sexual dimorphism in *Aedes scapularis* (Diptera: Culicidae). Biota Neotrop.

[12] Benítez HA, Parra LE, Sepulveda E, Sanzana MJ (2011). Geometric perspectives of sexual dimorphism in the wing shape of Lepidoptera: the case of *Synneuria* sp. (Lepidoptera: Geometridae). J Entomol Res Soc.

[13] Alexander RD, Brown W (1963). Mating behavior and the origin of insect wings.

[14] Shevtsova E, Hansson C, Janzen DH, Kjaerandsen J (2011). Stable structural color patterns displayed on transparent insect wings. PNAS Early Edition.

[15] Klingenberg CP, Debat V, Roff DA (2010). Quantitative genetics of shape in cricket wings: developmental integration in a functional structure. Evolution.

[16] Sperança MA, Capurro ML (2007). Perspectives in the control of infectious diseases by transgenic mosquitoes in the post-genomic era – a review. Mem Inst Oswaldo Cruz.

[17] Scali C, Catteruccia F, Li Q, Crisanti A (2005). Identification of sex-specific transcripts of the *Anopheles gambiae* double sex gene. J Exp Biol.

[18] Dujardin JP (2008). Morphometrics applied to medical entomology. Infect Genet Evol.

[19] Henry A, Thongsripong P, Fonseca-Gonzalez I, Jaramillo-Ocampo N, Dujardin JP (2010). Wing shape of dengue vectors from around the world. Infect Genet Evol.

[20] Leemingsawat S, Thongrungkiat S, Apiwathnasorn C, Singhaniyom S, Bellec C, N. J (2007). Influence of larval density or food variation on the geometry of the wing of *Aedes (Stegomyia) aegypti*. Trop Med Int Health.

[21] Kaba D, Ravel S, Acapovi-Yao G, Solano P, Allou K, Bosson-Vanga H (2012). Phenetic and genetic structure of tsetse fly populations (*Glossina palpalis palpalis*) in southern Ivory Coast. Parasit Vectors.

[22] Klingenberg CP (2010). Evolution and development of shape: integrating quantitative approaches. Nat Rev Genet.

[23] Lorenz C, Marques TC, Sallum MA, Suesdek L (2012). Morphometrical diagnosis of the malaria vectors *Anopheles cruzii.* An. homunculus and An. bellator. Parasit Vectors.

[24] Monteiro LR, Reis SF (1999). Princípios de morfometria geométrica.

[25] Motoki MT, Suesdek L, Bergo ES, Sallum MA (2012). Wing geometry of *Anopheles darlingi* Root (Diptera: Culicidae) in five major Brazilian ecoregions. Infect Genet Evol.

[26] Rohlf FJ (1993). Relative warp analysis and example of its application to mosquito wing.

[27] Vicente JL, Sousa CA, Alten B, Caglar SS, Falcuta E, Latorre JM (2011). Genetic and phenotypic variation of the malaria vector *Anopheles atroparvus* in southern Europe. Malar J.

[28] Vidal PO, Carvalho E, Suesdek L (2012). Temporal variation of wing geometry in *Aedes albopictus*. Mem Inst Oswaldo Cruz.

[29] Vidal PO, Suesdek L (2012). Comparison of wing geometry data and genetic data for assessing the population structure of *Aedes aegypti*. Infect Genet Evol.

[30] Vidal PO, Peruzin MC, Suesdek L (2011). Wing diagnostic characters for *Culex quinquefasciatus* and *Culex nigripalpus* (Diptera, Culicidae). Revista Brasileira Entomol.

[31] Demari-Silva B, Suesdek L, Sallum MAM, Marrelli MT (2014). Wing geometry of *Culex coronator* (Diptera: Culicidae) from South and Southeast Brazil. Parasit Vectors.

[32] Lorenz C, Marques TC, Sallum MAM, Suesdek L (2014). Altitudinal population structure and microevolution of the malaria vector *Anopheles cruzii* (Diptera: Culicidae). Parasit Vectors.

[33] Morais SA, Moratore C, Suesdek L, Marrelli MT (2010). Genetic-morphometric variation in *Culex quinquefasciatus* from Brazil and La Plata. Argentina. Mem Inst Oswaldo Cruz.

[34] Armell JH. A review of the *Scapularis* group of *Aedes* (*Ochlerotatus*), vol. 13. Contri Amer Ent*.* 1976.

[35] Consoli RAGB, Lourenço-de-Oliveira R (1994). Principais mosquitos de importância sanitária no Brasil. [online].

[36] Lane J (1953). Neotropical culicidae.

[37] Zavortink TJ (1973). Mosquito studies (Diptera: Culicidae) XXIX. A review of the subgenus *Kerteszia* of *Anopheles*. Contri Amer Ent.

[38] Rohlf FJ (1999). Shape statistics: procrustes superimpositions and tangent spaces. J Classif.

[39] Klingenberg CP (2011). MORPHOJ: an integrated software package for geometric morphometrics. Mol Ecol Resour.

[40] Felsenstein J. PHYLIP (Phylogeny Inference Package). 3.6 edn. University of Washington, Seattle: Distributed by the author. Department of Genome Sciences; 2005

[41] Dujardin JP (2010). COO, MOG and COV for windows.

[42] Dworkin I, Gibson G (2006). Epidermal growth factor receptor and transforming growth factor-b signaling contributes to variation for wing shape in *Drosophila melanogaster*. G3 (Bethesda).

[43] Klingenberg CP, Zaklan SD (2000). Morphological integration between developmental compartments in the *Drosophila* wing. Evolution.

[44] Gilchrist AS, Azevedo RBR, Partridge L, O’Higginsc P (2000). Adaptation and constraint in the evolution of *Drosophila melanogaster* wing shape. Evol Dev.

[45] Caprio MA, Huang JX, Faver MK, Moore A (2001). Characterization of male and female wing-beat frequencies in the *Anopheles quadrimaculatus* complex in Mississippi. J Am Mosq Control Assoc.

[46] Robertson SP, Caprio MA, Faver MK (2002). Heritability of wing-beat frequency in *Anopheles quadrimaculatus*. J Am Mosq Control Assoc.

[47] Cepeda-Pizarro J, Vásquez H, Veas H, Colon G (1996). Relaciones entre tamaño corporal y biomasa en adultos de *Tenebrionidae* (*Coleoptera*) de la estepa costera del margen meridional del desierto chileno. Rev Chil Hist Nat.

[48] Abbott JK, Bedhomme S, Chippindale AK (2010). Sexual conflict in wing size and shape in *Drosophila melanogaster*. J Evol Biol.

[49] Reeve JP, Fairbairn DJ (1999). Change in sexual size dimorphism as a correlated response to selection on fecundity. Heredity.

[50] Moller AP, Zamora-Muñoz C (1997). Antennal asymmetry and sexual selection in a cerambycid beetle. Anim Behav.

[51] Forrest TG (1987). Insect size tactics and developmental strategies. Oecologia.

[52] Allen CE, Zwaan BJ, Brakefield PM (2011). Evolution of sexual dimorphism in the Lepidoptera. Annu Rev Entomol.

[53] McAlpine DK (1973). Observations on sexual behavior in some Australian Platystomatidae (Diptera: Schizophora). Rec Aust Mus.

[54] Sivinski J (1997). Ornaments in the Diptera. Fla Entomol.

[55] Chenoweth SF, Rundle HD, Blows MW (2008). Genetic constraints and the evolution of display trait sexual dimorphism by natural and sexual selection. Am Nat.

